# Eliminate Dynamic Error of A-PNAS High-Precision Time Synchronization Using Multi-Sensor Combination

**DOI:** 10.3390/s25196028

**Published:** 2025-10-01

**Authors:** Zhenling Wang, Haihong Tao, Fang Hao, Yilong Liu, Zhengyong Wang

**Affiliations:** 1 National Key Laboratory of Radar Signal Processing, Xidian University, Xi’an 710071, China; wangzlcti@163.com (Z.W.); hhtao@xidian.edu.cn (H.T.); 2 The 54th Research Institute of China Electronics Technology Group Corporation, Shijiazhuang 050081, China; lylogn@126.com (Y.L.); 15373849955@163.com (Z.W.)

**Keywords:** A-PNAS, time synchronization, dynamic error, multi-sensor combination, clock skew

## Abstract

High-precision time synchronization among nodes of the airborne-based pseudolite navigation augmentation positioning system (A-PNAS) is a crucial indicator for ensuring the accuracy of positioning services. Due to the flight characteristics and external factors’ influence, the airborne platform usually undergoes random motion. Therefore, the time-varying effect errors and Doppler effect errors will be introduced into the clock skew measurement results during the time-synchronous processing. In A-PNAS with meter-level positioning accuracy, the time synchronization accuracy (TSA) between nodes usually needs to be within 2 ns. These dynamic errors will have an impact on the TSA between nodes, which cannot be ignored. Based on the analysis of the principle of dynamic error generation and the available sensors, a multi-sensor combination method for correcting dynamic errors is proposed. This method calculates and corrects the dynamic errors based on the motion measurements from sensors. The simulation test results show that the degree of improvement in correcting dynamic errors by this method is basically close to 80%. It can effectively meet the requirements of high-precision time synchronization system and can provide an effective reference for the high-precision time synchronization processing of similar space-based platform collaborative systems.

## 1. Introduction

With the rapid development and wide application of satellite navigation technology, pseudolite navigation augmentation systems (PNAS) and related technologies have received considerable attention [1,2,3]. PNAS can provide enhanced services and maintain positioning continuity when ground navigation receivers are denied or partially denied satellite signals, thus having significant application potential in disaster relief and military applications [4,5]. Airborne pseudolite navigation augmentation systems (A-PNAS) have attracted more attention due to their large coverage area and easy establishment of visual links. However, the random movement of platforms in A-PNAS makes high-precision time synchronization challenging, which is a key technology for high-precision positioning services. Meanwhile, with the rapid development of unmanned aerial vehicle (UAV) application technology, UAVs have been widely used in military reconnaissance and strike, disaster relief, emergency communication, and fire rescue, among others [6,7]. In scenarios where multiple UAVs need to work collaboratively and provide services, the high-precision time synchronization between UAVs is an important problem that needs to be solved.

In multiple airborne node cooperative systems represented by A-PNAS, it is necessary to achieve precise synchronization of all nodes and establish a unified time reference [8,9,10]. When the requirement of the TSA for platforms is general, the time synchronization can be completed by directly using the timing function of Global Navigation Satellite System [11]. At this time, there is no need for complex information interaction between platform nodes, and the accuracy of time synchronization generally ranges from 20 ns to several tens of ns. For the A-PNAS which provides precise positioning services, the required TSA is typically 10 nanoseconds or higher [12]. For scenarios demanding meter-level positioning accuracy, the TSA between nodes usually needs to be better than 2 ns. Take the typical ground-based pseudo-satellite positioning system Locata as an example, its TSA can reach 1 ns [13], and it can thereby provide high-precision regional positioning services.

Compared with the auxiliary synchronization method (ATSM) [11], the node independent synchronization method (ITSM) is more suitable for scenarios requiring higher precision. The specialized equipment and protocols are used by ITSM to achieve the time synchronization between system nodes. Unidirectional time synchronization and bidirectional time synchronization are two typical ITSMs [14], among which bidirectional time synchronization is more often applied for higher TSA [14,15,16]. The use of NTP (Network Time Protocol) and PTP (Precision Time Protocol) represents two of the most common bidirectional time synchronization methods. Under the assumption that the forward and backward delays of the bidirectional time synchronization signal are equal, PTP can achieve nanosecond-level TSA [15,16]. In the actual working scenarios, the random movement of airborne platforms will cause changes in the round-trip delay, and it is difficult to meet the TSA requirements of A-PNAS by directly using PTP.

Compared with the working mode of multiple airborne platforms, the measurement of inter-satellite two-way clock offset is a scenario that can be appropriately referred to. Researchers have conducted some research on the problem of time synchronization errors caused by satellite motion, and have proposed some methods for error compensation or accuracy improvement suitable [17,18,19]. These methods usually require prior knowledge of satellite motion. The movement of the airborne platform is more random and flexible due to the influence of external factors, and it is difficult to obtain prior information of the movement. Reference [20] proposed an improved dual one-way ranging measurement UAV time synchronization method, which achieved a TSA of 10 ns. Reference [21] proposed a multi-stage compensation bidirectional time synchronization algorithm for UAV cluster. Reference [22] proposed a nanosecond-level time synchronization experimental system based on a 2.4 GHz long-distance wireless link. These methods have a significant reference scenario, but none of them conduct targeted research on the dynamic errors introduced by random movements and the corresponding correction measures. Reference [23] proposed a dual-triggered bidirectional time synchronization method for unmanned aerial vehicle platforms in cases of uniform motion, and verified the effectiveness of it, but the achievable accuracy was only approximately 40 ns. Due to the limitations such as the lack of prior information, insufficient dynamic consideration, or limitations of accuracy, these methods cannot meet the TSA requirements for the A-PNAS which are used for meter-level positioning.

This article begins by discussing the requirements scenarios for high-precision time synchronization and the principle of bidirectional time synchronization in Section 2, as well as the principle and impact of dynamic being thoroughly analyzed. Then, Section 3 constructed the dynamic error mode, and clarified the observed quantity of motion state for eliminating dynamic errors. Section 4 proposed the suggestion for the selection of applicable sensors based on the performance comparison, and the residual errors for the dynamic error correcting using observation data form sensors has also been analyzed. Section 5 presented the preprocessing method for the sensor motion state observation data and the processing flow for eliminating the dynamic error of time synchronization through the combination of multiple sensors. Finally, a verification experiment on dynamic error correction was conducted using a combination of software simulation and hardware testing. The test results of this experiment were also explained and analyzed in Section 5. Compared with other methods, the method proposed in this article does not require prior information about the motion state and does not increase the equipment cost additionally. Due to its better precision, this method is more suitable for real-time elimination of the dynamic error of A-PNAS‘s airborne platforms.

## 2. Analysis of Dynamic Error Impact in Time Synchronization of A-PNAS

### 2.1. High-Precision Time Synchronization Requirements of A-PNAS

**Figure 1:**
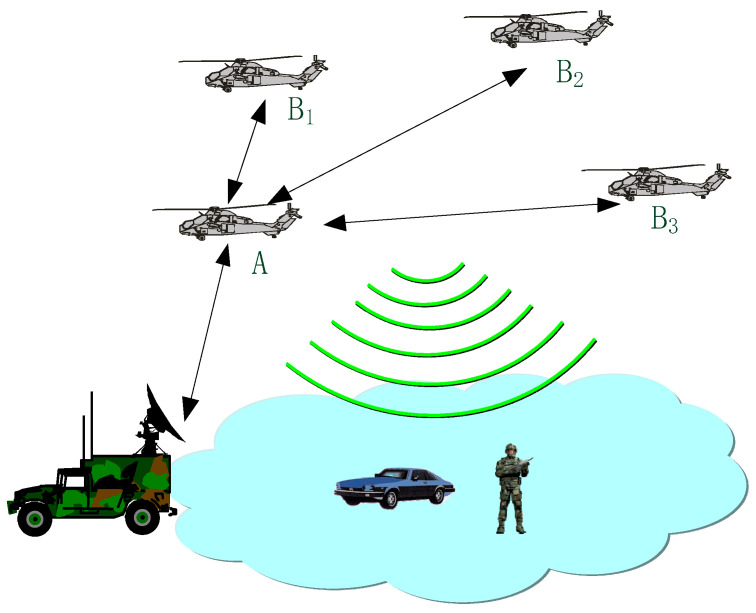
Schematic diagram of typical A-PNAS.

In A-PNAS, one ground central control vehicle serves as the anchor point, and the airborne platform, which composed of no fewer than four helicopters or unmanned aerial vehicles (UAVs), assumes the role of simulated satellites and transmits enhanced signals for positioning. The schematic diagram of the composition of the A-PNAS system was provided in Figure 1. This pseudo-satellite system can provide positioning service, and can also achieve coordinated work with the actual satellite constellation.

The ground control center provides time and space reference for the entire system. Considering the coverage area and the feasibility of line-of-sight communication, the nearest airborne platform A is selected as the main node. High-precision time synchronization is completed between control center and node A for transfer the time reference to the other slave nodes. The remaining airborne platform nodes B1–B3 are slave devices and each of them realizes time synchronization and information interconnection with the main node through wireless links. In order to maintain the system’s sufficient positioning accuracy, high-precision time synchronization must be maintained between the airborne platforms.

### 2.2. Bidirectional Time Synchronization Between Airborne Platforms

**Figure 2:**
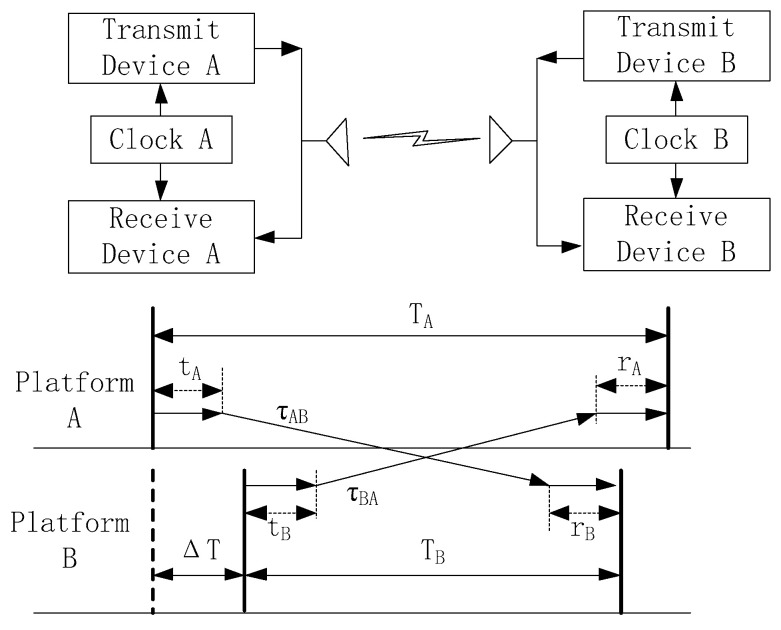
Principle of Bidirectional Time Synchronization Clock Skew Measurement.

The principle of bidirectional time synchronization between the airborne platforms is illustrated in Figure 2. ∆T is the measurements of the clock skew between platform A and platform B. The pseudo-range measurement values of platform A and platform B is $T_{A}$ and $T_{B}$. $t_{A}$ and $t_{B}$ denote the inherent processing delays of the transmitting devices A and B, respectively, and $r_{A}$, $r_{B}$ is the inherent processing delays of the receiving devices A and B. Additionally, the propagation time delay of the time synchronization signal from the transmit device A to the receive device B is $\tau_{AB}$, while the same delay from B to A is $\tau_{BA}$.

The delay values $T_{A}$ and $T_{B}$ can be expressed as:

(1) $$T_{A}=∆T+t_{B}+\tau_{BA}+r_{A}$$

(2) $$T_{B}=-∆T+t_{A}+\tau_{AB}+r_{B}$$

The clock skew ∆T can be obtained as follows:

(3) $$∆T=\frac{1}{2}\left(T_{A}-T_{B}\right)+\frac{1}{2}\left(\tau_{AB}-\tau_{BA}\right)+\frac{1}{2}\left(t_{AB}-t_{BA}\right)-\frac{1}{2}\left(r_{A}-r_{B}\right)$$

Inherent processing delays, such as $t_{A}$, $t_{B}$, $r_{A}$, and $r_{B}$, can usually be calibrated and corrected in advance. The Equation (3) can be further simplified to:

(4) $$∆T=\frac{1}{2}\left(T_{A}-T_{B}\right)+\frac{1}{2}\left(\tau_{AB}-\tau_{BA}\right)$$

After correcting the system inherent processing errors, the accuracy of the clock skew between platforms mainly depends on the pseudo-range difference $\frac{1}{2}\left(T_{A}-T_{B}\right)$ and the signal propagation delay difference $\frac{1}{2}\left(\tau_{AB}-\tau_{BA}\right)$.

If the relative positions or distances of platforms A and B remain unchanged during the measurement process, it can be considered that the time delays $\tau_{AB}$ and $\tau_{BA}$ for bidirectional signal transmission are equal, and the time synchronization skew can be calculated directly using the observation values $T_{A}$ and $T_{B}$ of the platforms. However, there are many uncertain factors in the state of the airborne platform while it is flying in the air, such as autonomous attitude and position control, and external physical factor disturbances, which can cause random relative movements between platforms. It is necessary to analyze and correct the time synchronization error caused by the relative motion for higher TSA.

### 2.3. Analysis of Dynamic Errors and Their Impact

The random relative motion between airborne platforms mainly manifests as approaching or moving away from each other. When using the bidirectional time synchronization processing method, the impact of motion on time synchronization can only consider the scenarios of approaching or moving away. According to the principle and process of time delay measurement, it can be known that due to the existence of random movement, the measurement results will be affected by the change in the distance between the transmitting and receiving devices at the observation moment, as well as the signal Doppler effect. Essentially, random motion causes the position change of the platform during the transmit time of the signal. This positional change is equivalent to causing a change in the observation moment of the receiving platform, which can be called time-varying effect error (TEE). For the transmission of the time synchronization signal, the relative movement will cause the relativistic Doppler effect. The Doppler effect of the signal will change the calculation dimension of the delay measurement and introduce a deviation in the measurement value, which can be called the Doppler effect error (DEE).

#### 2.3.1. The Time-Varying Effect Error Caused by Motion

**Figure 3:**
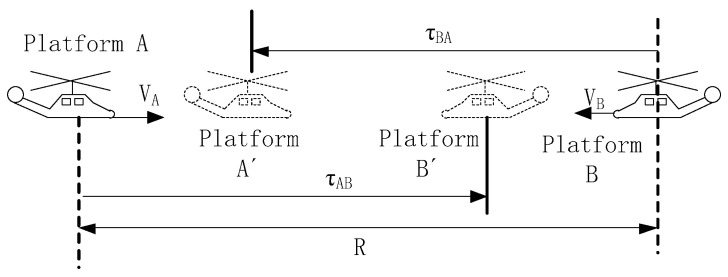
The Influence of Time-varying Effects on The Measurement of Clock Skews.

Assume that the initial distance between the airborne platforms A and B is R, and their relative movement mode is towards each other in a straight line. Figure 3 presents a schematic diagram illustrating the impact of opposite-direction motion under this condition on the clock skew measurement. Due to the change in the position of the receiving platform within the propagation delay of the signal, the observation moment of R measurement changes.

Let the radial motion velocities of platforms A and B be $V_{A}$ and $V_{B}$ respectively, and c represent the speed of light. The signal transmit moment of platform A and B can be regarded as the initial moment. The distance R can be expressed as:

(5) $$R=c\cdot \tau_{BA}+V_{A}\cdot \tau_{BA}$$

(6) $$R=c\cdot \tau_{AB}+V_{B}\cdot \tau_{AB}$$

From Equations (5) and (6), the time synchronization signal propagation delay from the signal transmission moment to the reception moment can be further expressed as follows:

(7) $$\tau_{BA}=\frac{R}{c+V_{A}}$$

(8) $$\tau_{AB}=\frac{R}{c+V_{B}}$$

It can be seen that the movement towards each other causes the position of platform change at the receiving moment compared to its initial position. The radial velocity will cause the signal propagation delay measured value to be smaller, and the deviation value is related to the motion speed of the receiving platform. Similarly, the signal propagation delay of the airborne platform is moving away from each other can be expressed as:

(9) $$\overset{\prime }{\tau_{BA}}=\frac{R}{c-V_{A}}=\frac{R}{c+\left(-V_{A}\right)}$$

(10) $$\overset{\prime }{\tau_{AB}}=\frac{R}{c-V_{B}}=\frac{R}{c+\left(-V_{B}\right)}$$

Uniformly use $V_{A}$ and $V_{B}$ to represent the radial relative motion velocities of platform A and B. By distinguishing the directions of motion using the sign of the values, the actual measured signal propagation delay can be uniformly represented by Equations (7) and (8).

Let the nominal value of the signal propagation delay under the condition of the platform is stationary be τ, which can be expressed using R:

(11) $$\tau =\frac{R}{c}$$

TEE can be expressed as the deviation between the measured values of platform A and platform B and their nominal values, which denoted as ${∆\tau }_{BA}$ and ${∆\tau }_{AB}$, respectively. They can be further expressed by the following formula:

(12) $${∆\tau }_{BA}=\tau_{BA}-\tau =\frac{R}{c+V_{A}}-\frac{R}{c}$$

(13) $${∆\tau }_{AB}=\tau_{BA}-\tau =\frac{R}{c+V_{B}}-\frac{R}{c}$$

**Figure 4:**
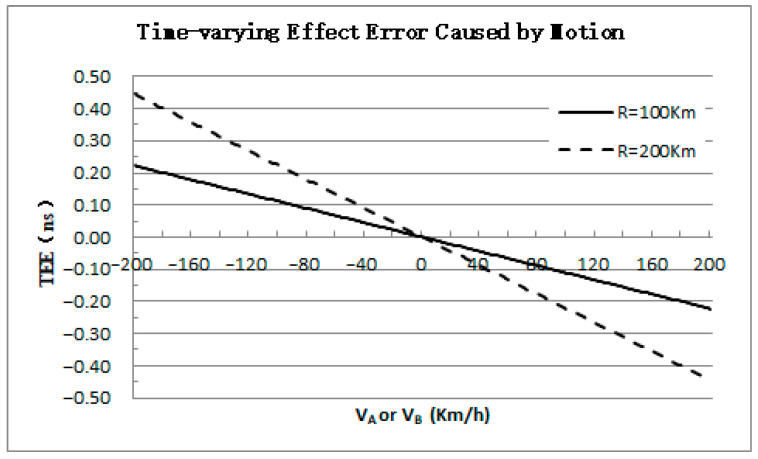
The TEE Varies with the Radial Speed of Platform.

The calculation of time synchronization is generally completed independently by the platform. One platform is selected to conduct independent analysis of dynamic TEE. In A-PNAS, considering the actual service area requirements, the distance between airborne platforms is usually between 100 and 200 km, and the movement speed is generally no more than 200 km/h. Taking c = 2.998 × 10^8^ m/s, Figure 4 illustrates the variation curves of ${∆\tau }_{BA}$ or ${∆\tau }_{AB}$ with the platform speed.

When moving towards each other, $V_{A}$ and $V_{B}$ are positive, and TEE is negative, resulting in a measurement value that is less than the nominal value; when moving away from each other, TEE is positive, and the measurement value will be greater than the nominal value. Taking the distance R = 200 km, the variation range of TEE can reach ±0.45 ns. Clearly, in the A-PNAS for meter-level positioning accuracy, this time-varying effect error should not be ignored.

#### 2.3.2. Relativistic Doppler Effect Error Caused by Motion

The time delay of the receiving equipment is mainly extracted through the signal phase between the transmission moment and reception moment, whose calculation unit is the chip width or carrier period of the signal. The relativistic Doppler effect caused by motion is equivalent to changing the unit of the measurement value calculation and will directly affect the measurement result. Taking the time synchronization signal parameters of a typical system as an example, which uses spread spectrum signals, the spread code rate is selected as 10 MHz and the center frequency of the signal is 1.5 GHz. When the motion speed is 200 km/h, the carrier Doppler caused by unilateral movement will reach 556 Hz, and the pseudo-code Doppler will also reach 3.7 Hz.

Taking platform A as an example, the Doppler of received signal is related to both the relative radial velocity of itself and that of platform B, and the Doppler effects of the two platforms A and B are the same. The motion velocity is much less than the speed of light, and the relativistic Doppler formula can be approximately expressed as [24]:

(14) $$f_{0A}^{\prime }=f_{0B}^{\prime }=f_{0}^{\prime }\approx f_{0}\cdot \left(1+\frac{V_{A}+V_{B}}{c}\right)$$

In this formula, $f_{0}$ denotes the nominal frequency of the time synchronization signal, $f_{0A}^{\prime }$ and $f_{0B}^{\prime }$ are the signal frequencies received of platforms A and B under the dynamic conditions, respectively, and they can be represented by a unified $f_{0}^{\prime }$. The Doppler frequency offset of the receiving device can be further expressed as:

(15) $$∆f=∆f_{A}=∆f_{B}=f_{0}^{\prime }-f_{0}=f_{0}\cdot \frac{V_{A}+V_{B}}{c}$$

Doppler can equivalently compress or stretch the pseudo-code rate or chip width used for pseudo-range measurement, and the increased or decreased measurement result will be caused inevitably if the changed chip width (or phase) is used for calculation. Further analysis shows that the proportion of the Doppler effect on the pseudo-code rate is the same as that on the carrier frequency. It can be considered that the calculation deviation $∆\tau_{A}$ and $∆\tau_{B}$ caused by platform Doppler is proportional to the Doppler frequency difference, and it can be further approximated using ∆τ as:

(16) $$∆\tau =∆\tau_{A}=∆\tau_{B}=\tau \cdot \frac{∆f}{f_{0}}$$

Substituting (11) and (15) into Equation (16), we can further obtain:

(17) $$∆\tau =∆\tau_{A}=∆\tau_{B}=\tau \cdot \frac{{∆f}_{A}}{f_{0}}=\frac{R}{c}\cdot \frac{V_{A}+V_{B}}{c}=\frac{R\left(V_{A}+V_{B}\right)}{c^{2}}$$

**Figure 5:**
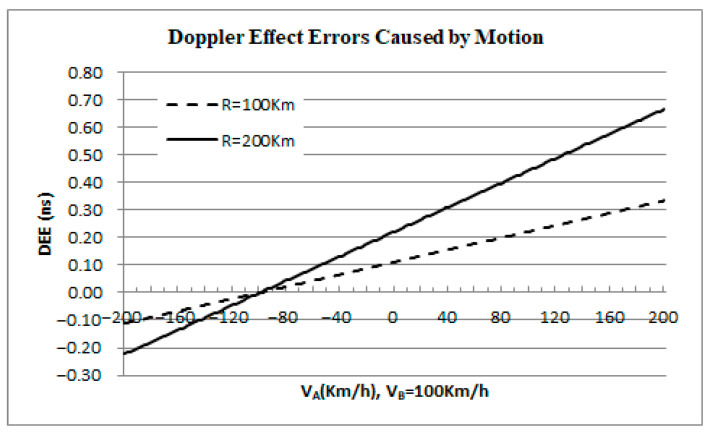
The Influence of Platform Radial Velocity on DEE.

Assume that platforms A and B are moving towards each other. Platform A has a variable velocity and the speed $V_{A}$ ranges from ±200 km/h, while platform B moves at a constant speed of 100 km/h. Setting the distance to 100 km and 200 km, respectively, substituting it into Equation (17), the curves of Doppler effect error $∆\tau_{A}$ or $∆\tau_{B}$ that varies with the radial velocity of platform A are shown in Figure 5.

According to the calculation, when the distance is 200 km and platform A moves away from platform B at a speed of 200 km/h, the DEE is at its minimum value, which is approximately 0.22 ns. When the two platforms move towards each other and the speed of platform A reaches a maximum of 200 km/h, the DEE will reach 0.67 ns. Clearly, in the A-PNAS for meter-level positioning accuracy, the DEE should not be ignored either.

## 3. Dynamic Error Model of Time Synchronization

**Figure 6:**
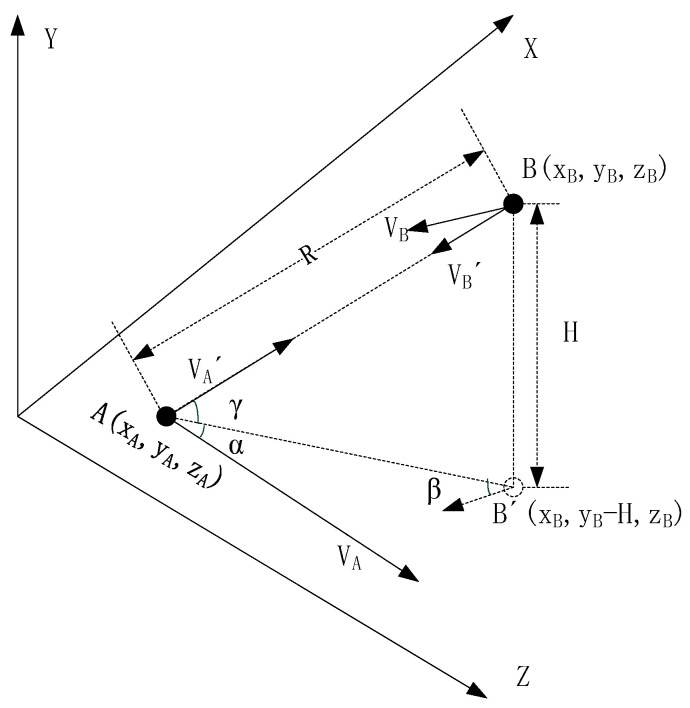
The Relative Motion of Platforms A and B in a Unified Coordinate System.

According to the abovementioned analysis, the dynamic errors TEE and DEE are mainly related to the distance between platforms and the relative radial velocity. The elimination of the error requires first obtaining R and $V_{A}^{\prime }$ and $V_{B}^{\prime }$ through external means. In order to be consistent with the data system of the sensor devices installed on the platform, place the airborne platform of A-PNAS into the geocentric coordinate system for analysis, such as the CGCS200. The relative motion of platform A and platform B in the coordinate system is presented in Figure 6, where the XY plane of the coordinate system coincides with the Earth’s equatorial plane, the *X*-axis points towards the 0-longitude direction, and the *Z*-axis points towards the Earth’s North Pole.

The initial position coordinates of platforms A and B in the figure are ($x_{A}$, $y_{A}$, and $z_{A}$) and ($x_{B}$, $y_{B}$, and $z_{B}$), and it is assumed that both platforms move horizontally with velocities $V_{A}$ and $V_{B}$, respectively. The movement direction of platform A is towards the north, consistent with the direction of the *Z*-axis; $B^{\prime }$ is the projection of platform B on the plane of platform A’s movement. Let the angle between the $V_{A}$ direction and the line segment A$B^{\prime }$ be α, and the angle between the $V_{B}$ direction and the line segment A$B^{\prime }$ be β. Considering the potential influence of the height difference between the platforms, let the elevation angle of platform A towards platform B be γ, and the height difference between the two be H. Then, the radial velocity of the two platforms moving towards each other can be expressed as:

(18) $$V_{A}^{\prime }=V_{A}\cdot cos\alpha \cdot cos\gamma$$

(19) $$V_{B}^{\prime }=V_{B}\cdot cos\beta \cdot cos\gamma$$

The γ can be further expressed by H and R:

(20) $$\gamma =arcsin\frac{H}{R}$$

The H can generally be obtained by subtracting $H_{A}$ from $H_{B}$, where the $H_{A}$ and $H_{B}$ are measurements from height sensors on the platforms:

(21) $$H=H_{A}-H_{B}$$

The R can be calculated using the coordinate data obtained from the position sensors through the following formula:

(22) $$R=\sqrt{{\left(x_{A}-x_{B}\right)}^{2}+{\left(y_{A}-y_{B}\right)}^{2}+{\left(z_{A}-z_{B}\right)}^{2}}$$

Substituting (21) and (22) into Equation (20) yields:

(23) $$\gamma =arcsin\frac{H_{A}-H_{B}}{\sqrt{{\left(x_{A}-x_{B}\right)}^{2}+{\left(y_{A}-y_{B}\right)}^{2}+{\left(z_{A}-z_{B}\right)}^{2}}}$$

**Figure 7:**
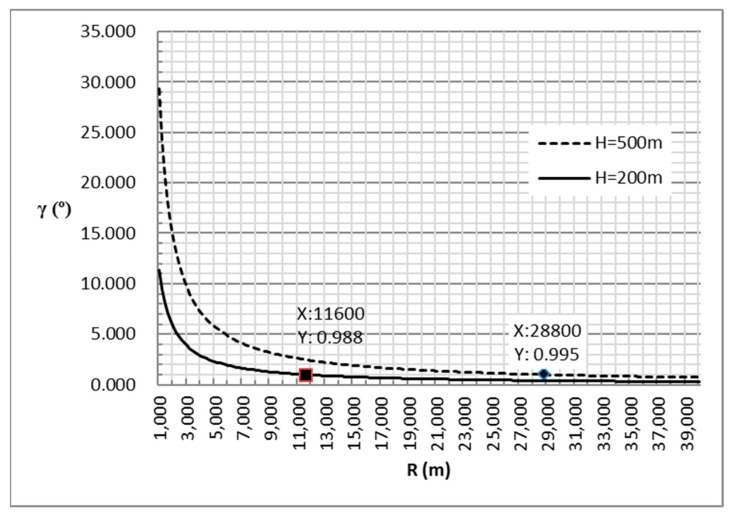
The Variation of Elevation Angle Under Height Restrictions with Distance.

In fact, the airborne platform of A-PNAS generally flies within an altitude range of 2 to 6 km, and the platform’s flight altitude is controlled to be as close as possible. The current technological means can effectively control the altitude difference between platforms within 200 m, and the maximum difference will not exceed 500 m. Assuming that the $y_{A}$ and $y_{B}$ coordinates in the position coordinates remain unchanged, the distance variation is only introduced by the change in the *X*-axis coordinate of the platforms, the curve of γ varying with the relative distance is shown in Figure 7.

In this result, when the altitude difference is 200 m and the distance greater than 11 km, the elevation angle can be lower than 1 degree. When the altitude difference is 500 m and the distance greater than 28.8 km, the angle is also below 1 degree.

Taking into account the influence of the service coverage area and the effect of signal near–far effect, the distance of the airborne platform of A-PNAS is generally no less than 20 km. When the height difference is 200 m, γ is only 0.6 degrees, and cosγ = 0.9999. Even if the height difference reaches 500 m, the angle is only 1.4 degrees, and cosγ=0.9997. It can be seen that the influence of the angle on the radial velocity can be ignored. Therefore, this paper will no longer consider the influence of elevation information in A-PNAS on the calculation of radial velocity. The Equations (18) and (19) can be simplified to Equations (24) and (25), which can be used for error calculation.

(24) $$V_{A}^{\prime }\approx V_{A}\cdot cos\alpha$$

(25) $$V_{B}^{\prime }\approx V_{B}\cdot cos\beta$$

By substituting $V_{A}^{\prime }$ and $V_{B}^{\prime }$ for $V_{A}$ and $V_{B}$ in Formulas (12), (13), and (17), the calculation formulas of TEE and DEE in the unified coordinate system of the platform sensors can be obtained:

(26) $$∆\tau_{BA}^{\prime }=\frac{R}{c+V_{A}cos\alpha }-\frac{R}{c}$$

(27) $$∆\tau_{AB}^{\prime }=\frac{R}{c+V_{B}cos\beta }-\frac{R}{c}$$

(28) $$∆\tau^{\prime }=∆\tau_{A}=∆\tau_{B}=\frac{R\left(V_{A}cos\alpha +V_{B}cos\beta \right)}{c^{2}}$$

By leveraging the various sensors provided by the platform, information such as horizontal movement speed, coordinate position, and movement azimuth angle can be obtained, which can be used for the calculation of dynamic errors. Then, using the calculation results, the clock skew in the time synchronization processing can be directly corrected, thereby achieving the goal of eliminating TEE and DEE in A-PNAS.

## 4. Analysis of Dynamic Error Estimation Accuracy and Correction Residuals

### 4.1. Selection of Velocity Measurement Sensors and Estimation Accuracy of Related Dynamic Error

As a standard configuration, the airborne platform is generally equipped with ground speed measurement sensor or equipment. Speed measurement is usually carried out mainly using GNSS, and INS is used as the auxiliary means. The measurement data obtained are provided to the time synchronization algorithm through simple interfaces. When the GNSS receiver experiences temporary instability, the INS can be used for providing velocity data, which can maintain the measurement accuracy for a certain period of time.

The GNSS receiver technology is highly mature and its speed measurement accuracy could be reached within 0.2 m/s [18]. For a maximum speed of 200 Km/h, this speed measurement deviation is only 0.36% of the speed value. Under this speed measurement accuracy, referring to Equations (12) and (18), the TEE and DEE could be corrected at least 99% without considering the deviation of the direction angle. The correction residual $\varepsilon_{V-TEE}$ will not exceed 1% of ${∆\tau }_{BA}$ or ${∆\tau }_{AB}$, which was about 0.005 ns, and the correction residual $\varepsilon_{V-DEE}$ of DEE will also not exceed 0.007 ns.

The basic principle of using inertial sensors for velocity measurement is to calculate the speed through the integration of the sensor. Due to the existence of the accumulation effect, inertial systems generally require GNSS for periodic calibration. The calibration interval or calibration period of INS can be regarded as the time required to maintain measurement accuracy. Inertial sensors can be classified into four levels, namely Navigation Grade, Tactical Grade, Industrial Grade, and Consumer Grade. Based on the product parameters of multiple companies such as Honeywell, Sagem, ST Microelectrocics, and Bosch Sensortec GnbH, the typical velocity measurement accuracy and positioning accuracy of different levels of inertial equipment can be sorted out and list in Table 1.

In A-PNAS, the airborne platforms usually select tactical-level inertial navigation products as the standard configuration, which can maintain a velocity measurement accuracy of better than 1 m/s within 10 h. By using the data from inertial sensors to correct the residuals $\varepsilon_{V-TEE}^{\prime }$ of TEE and $\varepsilon_{V-DEE}^{\prime }$ of DEE, they will not exceed 0.05 ns and 0.07 ns, respectively. When using industrial-grade inertial sensors in UAV, even in the case of a brief interruption of GNSS assistance, comparable correction accuracy can still be achieved. If there is a GNSS assistance interruption for a long time, the velocity measurement values of the industrial-grade inertial sensors will be difficult to meet the expected correction targets for TEE and DEE. For example, when the distance calculation deviation exceeds 400 m, the accuracy of error correction will deteriorate clearly, and even new errors may be introduced.

### 4.2. Selection of Relative Position Measurement Sensors and Estimation Accuracy of Related Dynamic Error

The position of airborne platform is generally obtained using GNSS. The positioning accuracy of GNSS, represented by the Beidou system, is shown in Table 2 [25].

According to the most basic performance parameter of the open signals of the Beidou system in Table 1, it can be seen that an ordinary airborne receiver can achieve a positioning accuracy of 15 m horizontally and 22 m in elevation in the worst case. According to Equation (23), it can be calculated that when the altitude of the platforms is similar, the influence of this positioning error on the distance is approximately 15 m at its maximum. Even if the altitude difference between the two platforms is close to 500 m, the influence of this positioning error on the distance is only about 16.4 m. According to Equations (12), (13) and (17), the value for the residuals $\varepsilon_{P-TEE}$ and $\varepsilon_{P-DEE}$ will not exceed 1 ps. It is denoted as $\varepsilon_{P-TEE}\approx 0$ and $\varepsilon_{P-DEE}\approx 0$, and which can be ignored.

### 4.3. Selection of Motion Angle Measurement Sensor and Estimation Accuracy of Related Dynamic Error

The heading angle of an aircraft is generally defined as the angle between the direction of the aircraft’s nose and the true north direction, and often a multi-sensor combination approach is employed to obtain this angle. For time synchronization processing with higher timeliness requirements, it is more suitable to directly adopt the real-time measurement data from a certain sensor. Based on the publicly available information, the main measurement methods and performance parameters of the heading angle can be summarized as shown in Table 3.

According to Equation (24), the DEE is proportional to the flight speed and the cosine value of the azimuth angle. When the angle measurement error is less than 5 degrees, the change in the cosine value does not exceed 0.087, which means that the residual error $\varepsilon_{A-DEE}$ will not exceed 8.7%, approximately 0.058 ns at the maximum. When the angle measurement error is less than 3 degrees, the residual error $\varepsilon_{A-DEE}$ will not exceed 5.2%, approximately 0.036 ns. According to Equations (22) and (23), the error in angle measurement is located in the denominator of the TEE calculation formula. Obviously, the influence coefficient on TEE is smaller and can be ignored, denoted as $\varepsilon_{A-TEE}\approx 0$.

Considering the actual engineering conditions and the acceptability of error correction, a measurement error of no more than 5 degrees in the azimuth angle is sufficient to meet the expected goals. Therefore, using an electronic compass is the preferred solution.

### 4.4. The Comprehensive Residual Error of Using Multiple Sensors to Correct Dynamic Errors

Based on the above analysis, using sensors with appropriate accuracy reasonably for obtaining motion speed, motion angle, and relative position measurements, the dynamic error TEE and DEE can be calculated and corrected. Under this sensor combination mode, the comprehensive correction residuals of TEE and DEE are:

$$\varepsilon_{DEE}=\sqrt{\varepsilon_{V-DEE}^{2}+\varepsilon_{P-DEE}^{2}+\varepsilon_{A-DEE}^{2}}=\sqrt{0.007^{2}+0.0^{2}+0.058^{2}}\approx 0.058\;ns$$

$$\varepsilon_{TEE}=\sqrt{\varepsilon_{V-TEE}^{2}+\varepsilon_{P-TEE}^{2}+\varepsilon_{A-TEE}^{2}}=\sqrt{0.005^{2}+0.0^{2}+0.007^{2}}\approx 0.009\;ns$$

When the GNSS is briefly affected, it is advisable to temporarily replace the GNSS with tactical-level inertial navigation system to obtain the location. The sensor combination of using the general GNSS receiver to measure the position and the electronic compass to measure the angle will still be the first selection. Due to the insensitivity to short-term position changes, the comprehensive correction residuals of TEE and DEE will be:

$$\varepsilon_{DEE}=\sqrt{{\varepsilon_{V-DEE}^{\prime }}^{2}+\varepsilon_{P-DEE}^{2}+\varepsilon_{A-DEE}^{2}}=\sqrt{0.07^{2}+0.0^{2}+0.058^{2}}\approx 0.09\;ns$$

$$\varepsilon_{TEE}=\sqrt{{\varepsilon_{V-TEE}^{\prime }}^{2}+\varepsilon_{P-TEE}^{2}+\varepsilon_{A-TEE}^{2}}=\sqrt{0.05^{2}+0.0^{2}+0.007^{2}}\approx 0.077\;ns$$

It can be seen that by using the aforementioned method to correct the dynamic error of the A-PNAS airborne platforms, the remaining residual error is generally no more than 0.1 ns. In terms of improvement degree, compared with the maximum value of 0.45 ns for TEE, the improvement degree can reach 89.6%; for the maximum value of 0.67 ns for DEE, the improvement degree can reach over 86%.

## 5. Data Preprocessing and Dynamic Error Correction Flow

### 5.1. Observation Data Preprocessing

Under the sensor combination method, the movement speed and position coordinates of the platform can be directly obtained, and they can be consistent with the coordinate system used in the aforementioned analysis. The observation data can be used directly after simple preprocessing such as removing unreasonable data and marking timestamps.

The calculation Formulas (25) to (27) for the aforementioned error calculation were all derived under the condition that the direction of the speed of platform A was consistent with the direction of the *Z*-axis. Let $\theta_{A}$ and $\theta_{B}$ denote the flight angle measurements obtained by platforms A and B using sensors, and their meanings are the angle between the flight direction and the true north or the direction of the *Z*-axis. They are not α and β in the formulas. Therefore, the flight angle measurements need to be transformed through preprocessing before they can be used for calculation.

**Figure 8:**
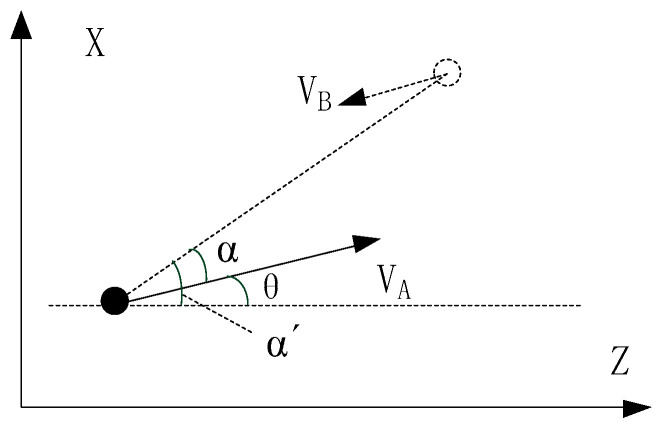
The Relationship Between Flight Angle and the Angle Error Calculation.

As shown in Figure 8, after obtaining the platform coordinates, simple geometric calculations using the coordinates within the XZ plane can be performed to obtain the angle α’ between the line segment AB’ in Figure 6 and the *Z*-axis (which represents true north). The calculation expression is:

(29) $$\alpha^{\prime }=arctan\frac{x_{B}-x_{A}}{z_{B}-z_{A}}$$

(30) $$\alpha =\alpha^{\prime }-\theta_{A}=arctan\frac{x_{B}-x_{A}}{z_{B}-z_{A}}-\theta_{A}$$

Similarly, the angle between the line segment AB’ and the *Z*-axis can be obtained as β’, and it is given as:

(31) $$\beta =arctan\frac{x_{B}-x_{A}}{z_{B}-z_{A}}-\theta_{B}$$

Through the above preprocessing, the angle data required for error calculation can be obtained.

### 5.2. Multi-Sensor Combination Dynamic Error Correction Processing Flow

**Figure 9:**
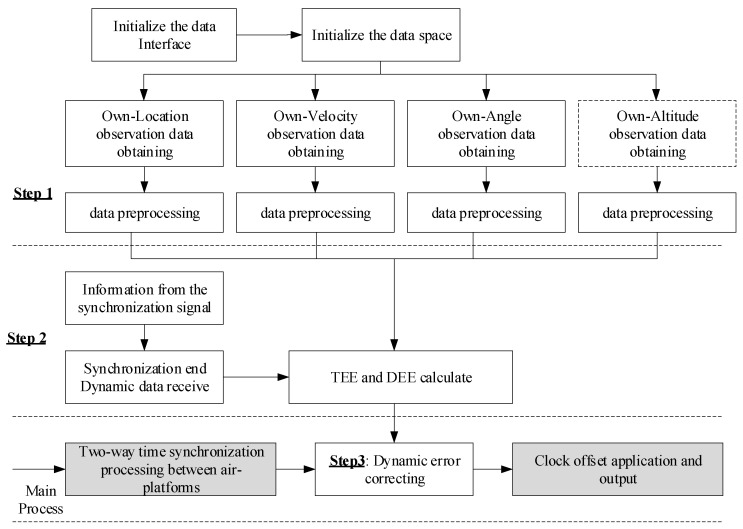
Dynamic Error Correction Processing Flow Utilize Multi-sensor Combination.

The dynamic error correction processing based on multi-sensor combination is incorporated into the bidirectional time synchronization processing flow as shown in Figure 9. Thus, the calculation and correction of TEE and DEE could be accomplished.

The entire processing flow can be divided into three steps:

Step 1: Measurements acquisition. After completing the initialization of the local data space and data interface, the original observational data such as position, speed, and azimuth angle are received through the data interface from the local sensors. In this step, these data undergo preprocessing and are transformed into the original data form that can be calculated according to Formulas (25) to (28).

Step 2: By extracting the information carried in the time synchronization signals, the dynamic data of the synchronous objects can be obtained. The dynamic errors TEE and DEE can be calculated by integrating the data of synchronous object and the self-data obtained in step one.

Step 3: The calculation results of TEE and DEE are incorporated into the main process of the time synchronization processing of the A-PNAS. Correct the dynamic errors of the obtained clock skew results, and the final time synchronization results are obtained.

### 5.3. Simulation Tests and Verification

Considering the difficulty of assessment and the numerous uncontrollable factors in the outdoor environment, a test ground verification environment was built using controllable conditions. The dynamic characteristics of the airborne platform were simulated by means of signal simulation, and the corresponding sensor simulation data were output. The applicability of the sensor simulation data has been recognized through its use in other tests and experiments.

**Figure 10:**
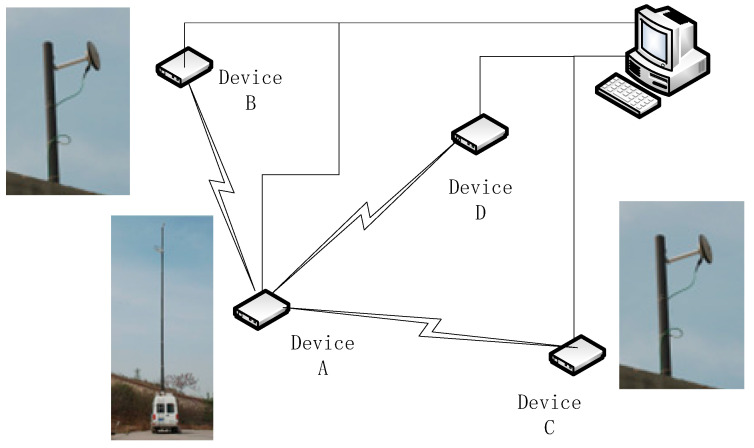
Simulation Experiment Verification Environment.

The experimental environment was shown in Figure 10. Four time-synchronization devices and the transmitting antenna were placed at the designated coordinates, with a physical distance of no less than 100 m. The broadcasting and adjustment control of all signals were completed by the management computer. Platform A was selected as the host. By controlling the inherent time delay of the signals, the initial distances between devices B, C, D, and A were all simulated to be 100 km, and the corresponding base coordinate simulation values of each device could be calculated.

In this experiment, each node used the same time synchronization device which runs the embedded software algorithms for signal generation and signal processing in the actual application. The control computer was used to control the four-node devices and analyze the respective time synchronization results. The time synchronization devices at each node generate time synchronization signals based on the local time reference, while simultaneously receiving and measuring the signal from the host node A. The time synchronization results were output to the control computer. The experiment only focused on the analysis and correction of dynamic errors. In order to eliminate the clock drift error between nodes, device A and other devices achieved initial time coarse synchronization through fibers, and the same reference frequency was used. The experiment adjusts the transmission delay of the signal through the pseudo-range simulation method, which can equivalently simulate a long signal transmission distance and the signal delay changes caused by platform movement.

To eliminate potential multipath effects, the antennas were raised during the experiment. The grassy condition of the dedicated test field also helps to reduce multipath effects. Since the physical layout of each device is actually fixed, even if there are multipath errors in the measured values, their values are relatively stable and can be processed as system errors. The dynamic parameters were converted into speed, acceleration, and coordinates by control computer, and which were transmitted to the node devices for completing the error calculation and correction in time synchronization processing.

Devices A, B, and C are in a stationary state and emit fixed and unchanging time synchronization signals. Device D is selected as the dynamic platform. In this case, the measurement values of the time synchronization deviation $∆T_{AB}$, $∆T_{AC}$, and $∆T_{AD}$ between Device A and the other three devices are compared, and the clock difference measurement results $∆T_{AD}$ and $∆T_{AD}^{\prime }$ before and after the dynamic error correction between Device A and Device D are given special attention.

**Figure 11:**
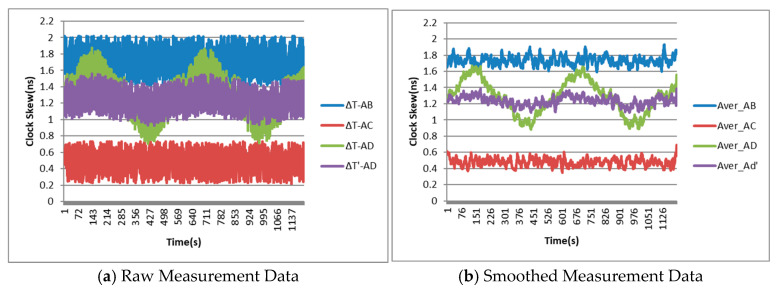
Clock Skew Measurement Results I.

Device D performs a uniform circular motion simulation with a radius of 5 km, and the movement speed is fixed at 200 km/h. Figure 11a presents the original experimental data for the clock skew over approximately 20 min. It can be seen that, compared with $∆T_{AB}$ and $∆T_{AC}$, the $∆T_{AD}$ exhibits two periodic fluctuations caused by the introduction of dynamic effects, which are consistent with the circular motion period of the device D. Moreover, after dynamic error correction, the fluctuation range of $∆T_{AD}^{\prime }$ becomes significantly smaller.

For the purpose of comparison, the original data in Figure 11a was simply smoothed and the data curve was plotted in Figure 11b. It can be seen that the fluctuation range of the smoothed data of $∆T_{AD}$ exceeded 0.7 ns, which was slightly larger than the error theoretical limit value in the aforementioned analysis. This is related to the actual experimental environmental factors to some extent. Moreover, by analyzing the smoothed data of $∆T_{AD}^{\prime }$, it can be seen that after dynamic error correction, the clock skew still has a slight fluctuation, but the fluctuation range has decreased to approximately 0.16 ns, with an improvement rate of about 77%, close to 80%.

Another experiment has been conducted with the motion simulation on all of the slave node devices. The parameter settings include:

(1) After the system started and operated for half an hour, the slave nodes B, C, and D started to enter the movement state.
(2) The slave node B performed an anticlockwise circular motion with a radius of 7 km and a speed of 200 km/h.
(3) The slave node C moved at a constant speed in a straight line with a speed of 150 km/h, making round trips within a range of ±20 km.
(4) The slave node D performed a clockwise circular motion with a radius of 5 km and a speed of 100 km/h.
(5) The process of movement simulation lasted for one hour, after which the distance remained at its final state.

**Figure 12:**
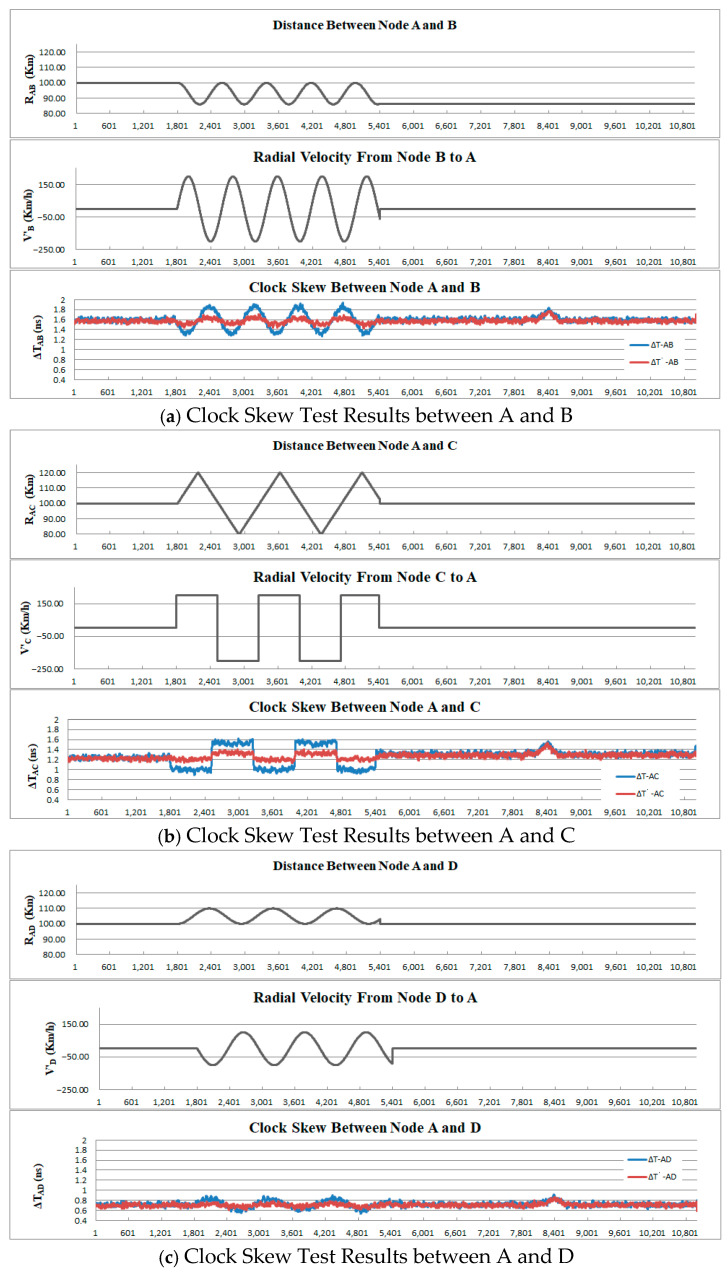
Clock Skew Measurement Results II.

The clock skew measurements between each slave node and node A are presented in Figure 12.

From this result, it can be seen that similar effects as those of the previous experiment have been achieved, and the dynamic errors have been significantly corrected. The clock skew of each node remained basically stable before and after the movement.

Examining the test results carefully, two additional phenomena were discovered. The clock offset $∆T_{AC}$ increased by approximately 0.1 ns after the motion simulation compared to before the motion, and this increase persisted until the end of the experiment. Since the dynamic error correction processing has stopped running, this phenomenon might be caused by other factors that affect the measurement of signal propagation delay. Furthermore, approximately 2 h and 20 min into the experiment, there was a simultaneous fluctuation of about 0.12 ns in the clock skews of all three nodes. This fluctuation is likely to stem from the common factors such as the wireless link environment or the device of node A.

### 5.4. Comparison with Existing Time Synchronization Methods

In the existing methods of time synchronization, attention has gradually been paid to the influence of dynamic errors. Compared with the traditional two-way time synchronization method, the dual-trigger two-way time synchronization method based on the PTP protocol [22] reduces the sensitivity to dynamic errors. However, this method has not further explored the targeted elimination of dynamic errors. Another method of improved dual one-way ranging measurement UAV time synchronization [20] is also similar to this. The system-level TSA targeted by these two methods is 10–20 nanoseconds, which is significantly different from the system accuracy requirements discussed in this paper. Reference [26] analyzed the relationship between TSA and distance and movement speed in the task coordination of swarm aircraft and proposed a method of using GNSS positioning data to correct dynamic errors. This method is very similar to the one proposed in this article. However, the TSA requirement for the system targeted by that method is 20 ns, which is still one order of magnitude lower than the requirement of A-PNAS.

Expanding the scope of related research, the time synchronization methods for inter-satellite links are also quite similar and have higher accuracy. The high accuracy physical time synchronization method based on two-way comparison [17] took into account the use of radial velocity prior information to correct the Doppler effect error, whose theoretical accuracy of eliminating dynamic errors is slightly better than the method in this article. Reference [18] analyzed and simulated the satellite motion time-varying effect error in inter-satellite time synchronization, and the correction accuracy was 0.2 ns, which is comparable to that of this paper. Both of the above two methods do not simultaneously take into account the time-varying effect error and the Doppler effect error. Moreover, satellite platforms usually have better prior information and no random motion.

Compared with these existing methods, the method proposed in this article takes into account both time-varying effect errors and Doppler effect errors simultaneously and has higher accuracy. Moreover, for airborne platforms, the multi-sensor combination method also has better adaptability and robustness.

## 6. Conclusions

The random motion of the airborne platform in A-PNAS will bring time-varying effect errors and Doppler effect errors. In response to the demand for meter-level positioning accuracy, it is very important to eliminate these errors in bidirectional time synchronization. The position, velocity, flight angle, and other data of platforms are obtained in real time through the multi-sensor combination. Using these data, the dynamic errors can be directly estimated and the time synchronization results can be corrected. The simulation test results show that the improvement degree of this method for dynamic errors can approach more than 80%, and the theoretical residual after dynamic error correction does not exceed 0.1 ns, which can provide a guarantee for the time synchronization performance of the meter-level position A-PNAS. Furthermore, this method can also provide an effective reference for the time synchronization processing of multiple airborne-based platforms collaborative systems.

In fact, the number of airborne platform nodes in A-PNAS is usually four to six, in which scenario the advantages of this method are quite obvious and applicable. If more synchronous nodes need to be added or cascaded, networking is required in other scenarios, directly using this method will lead to a significant increase on complexity parameter calculation. This necessitates further research on technologies for ad hoc networks and edge computing to improve and optimize the applicability of this method. Furthermore, the actual flight will also encounter problems such as multipath fading, radio frequency interference, and data packet loss in the wireless link. These issues pose challenges to the robustness and continuity of the system, which should be attention in future research.

## Figures and Tables

**Table 1:** Classification of Inertial Sensor Grades.

Garde	Positioning Accuracy	Rate Accuracy	Application
Navigation	≤0.1n mile (185 m) (in one hour)	≤0.01 m/s (in one hour)	Aircraft Inertial Navigation System Spacecraft orbit control
	≤1n mile (1.85 km) (in 10 h)	≤0.1 m/s (in 10 h)	
Tactical	≤1 Km (in one hour)	≤0.1 m/s (in one hour)	Drone navigation Ship combination navigation, Missile guidance
	≤10 m (GNSS assistant)	≤1 m/s (in 10 h)	
Industrial	≤10 m (in ten minutes)	≤0.5 m/s (in one minutes)	Industrial robot, UAV Trajectory planning (GNSS assistance needed)
	≥1 Km (after one hour)	≥30 m/s (after one hour)	
Consumer	≥10 m (be depended on GNSS or Wi-Fi)	≤5 m/s (in one second)	Personal attire Low costs UAV attitude assistance.
	—	≥50 m/s (after 10 s)	

**Table 2:** The Positioning Accuracy Standard.

Service Scheme	Positioning Accuracy Standard (95%)		Constraints
Single-frequency or dual-frequency	Average globally, Horizontal	≤9 m	The elevation mask is 5 degrees; Usage constraints are met, and healthy SISs are used for calculation; The statistical value of any 7-day positioning errors of all points in the global region; Excludes transmission errors and user segment errors.
	Average globally, Vertical	≤10 m	
Single-frequency or dual-frequency	Horizontal error at the worst point.	≤15 m	The elevation mask is 5 degrees; Usage constraints are met, and healthy satellite signals are used for calculation; The statistical value of any 7-day positioning errors of the world’s worst position; Excludes transmission errors and user segment errors.
	Vertical error at the worst point.	≤22 m	

**Table 3:** Measurement Methods and Performance of Platform Azimuth Angle.

Angel Measurement Sensors	Error	Advantage	Limitation
Magnetic Compass	Unable to Output	Simple	Extremely Susceptible to Interference
Electronic Compass	0.5°~2°	Easy to Integrate	Requires Regular Calibration
Strap-down Inertial Navigation System (SINS)	<0.1°/h (With Compensation); Without Compensation, the Accumulated Error over 10 h Amounts to Approximately 10°.	High Short-term Accuracy	Long-term Work Leads to Obvious Accumulation of Errors.
GNSS/INS Combination	Very Low (error < 0.1°)	High Precision	All-weather Operation, Relying on Satellite Signals
Doppler Radar	Medium (error 1°~3°)	Anti-obstruction	Affected by the Terrain The Equipment is Complex

## Data Availability

Due to privacy concerns, the data supporting the findings of this study are not publicly available.

## References

[1] Dou J., Xu B., Dou L. (2021). Impact Assessment of the Asynchronous Clocks Between Reference and User Receivers in Differential Pseudolite Navigation System. IEEE Sens. J..

[2] Gao H., Deng Z., Wang L., Shi L. (2021). Coverage Analysis of Air-based Regional Augmentation System for BD Satellite Navigation Signal. Radio Eng..

[3] Yu B., He C. (2017). Assessment of Air-ground Cooperation Pseudolites Augmentation System. J. Huazhong Univ. Sci. & Technol. (Nat. Sci. Ed.).

[4] Han P., Guo C., Li S., Yu J., Chen F. (2024). Emergency Communication Relay Deployment Based on UAV. Comput. Appl. Softw..

[5] Dong K., Xu W. (2023). Application Research and Development Trend of Firefighting Drone. Mod. Manuf. Technol. Equip..

[6] Xu Z., Wang B., Yun X., Wang X. (2022). Stable relay selection method under an uncertain preference ordinal for UAV in post-disaster. J. Xidian Univ..

[7] Zhao Q., Zhu C., Qian F., Yang Z., He W. (2023). Research on Application Development of Small and Medium-sized Shipborne UAVs on Ships. Ordnance Ind. Autom..

[8] Kabiri M., Cimarelli C., Bavle H., Sanchez-Lopez J.L., Voos H. (2023). A Review of Radio Frequency Based Localisation for Aerial and Ground Robits with 5G Future Perspectives. Sensors.

[9] Xue C., Li T., Li Y. (2024). Radio Frequency Based Distributed System for Noncooperative UAV Classification and Positioning. J. Inf. Intell..

[10] Ko S.-W., Chea H., Han K., Lee S., Seo D.-W., Huang K. (2021). V2X-Based Vehicular Positioning: Opportunities, Challenges, and Future Directions. Wirel. Commun..

[11] Seijo O., Val I., Luvisotto M., Pang Z. (2021). Clock synchronization for Wireless Time-Sensitiv Networking: A March from Microsecond to Nanosecond. IEEE Ind. Electron. Mag..

[12] Yang F., Fan J., Ma J., Ma Z., Wu X., Liao B. (2021). Time Syachronization Technology and Precision Analysis ov Navigation Enhanced Pseudolite. Navig. Psiotioning Timing.

[13] Chen J., Peng L., Huang Q. (2018). Time Synchronization Mechanism for Locata Positioning System. GNSS World China.

[14] Ming Z., Pang H., Xu Y., Zhang L., Zhang W. (2022). Estimating Clock Skew with One-Way Timestamps. IEEE Commun. Lett..

[15] Pranger S., Haynes M.S., Moghaddam M. (2020). Wireless Sub-nanosecond RF Synchronization for Distributed Ultrawideband Software-Defined Radar Networks. IEEE Trans. Microw. Theory Technol..

[16] Merlo J.M., Mghabghab A.R., Nanzer J.A. (2023). Wireless Picosecond Time Synchronization for Distributed Antenna Arrays. IEEE Trans. Microw. Theory Technol..

[17] Yu X., Wang D., Li Z., Zhao H. (2019). High Accuracy Physical Time Synchronization Method Based on Two-Way Comparison. Acta Aeronaut. Astronaut. Sin..

[18] Huang F., Chen Y., Li T., Shan Q., Zhang J., Cai C. (2019). Analysis and Correction to the Influence of Satellite Motion on the Measurement of Inter-Sallite Two-Way Clock Offset. EURASIP J. Wirel. Commun. Netw..

[19] Bai Y., Lu X., Gao T. (2021). An Improved Algorithm for Inter-Satellite Link Time Synchronization Based on Single-Point Paeudo-Rang Reduction. Geomat. Inf. Sci. Wuhan Univ..

[20] Liu G., Lei M. (2023). Research on an improved dual one-way ranging measurement UAV time synchronization method. Wirel. Internet Technol..

[21] Zhang R., Liu C., Cheng J., Ding Y. (2021). A Multi-Stage Compensation Bidirectional Time Synchronization Algorithm for UAV Cluster. Comput. Simul..

[22] Müller P., Berger D., Sarperi L. (2025). Nanosecond Time Synchronization over a 2.4 GHz Long-Range Wireless Link. Sensors.

[23] Chen C., Duan B., Xu Q., Pan W., Ma W., Shao S. (2024). High precision time synchronization between nodes under motion scenario of UAV platforms. J. Xidian Univ..

[24] Zha G., Xiong X. (1990). Spread-Spectrum Communication.

[25] China Satellite Navigation Office (2021). Beidou Navigation Satellite System Open Service Performance Stardard (Version 3.0).

[26] Shang Y., Zhang D., Wang L., Li Z., Li S. A High-precision Time Synchronization Algorithm for Aviation Swarm Task Cooperation. Proceedings of the IEEE 2nd International Conference on Electronic Technology, Communication and Information (ICETCI).

